# Development of a Nano‐Hybrid Composite Using Bovine Hydroxyapatite and Montmorillonite for Endodontic Applications—An In Vitro Study

**DOI:** 10.1111/iej.70163

**Published:** 2026-04-15

**Authors:** P. A. A. S. Prasad Kumara, Paul R. Cooper, Peter Cathro, Henry F. Duncan, Maree Gould, George Dias, Jithendra Ratnayake

**Affiliations:** ^1^ Sir John Walsh Research Institute, Faculty of Dentistry University of Otago Dunedin New Zealand; ^2^ Dublin Dental University Hospital, Trinity College Dublin University of Dublin Dublin Ireland; ^3^ Department of Anatomy, School of Medical Sciences University of Otago Dunedin New Zealand

**Keywords:** bioceramics, bovine hydroxyapatite, characterisation, composite, endodontics, hydraulic calcium silicate cements, montmorillonite

## Abstract

**Aim:**

Hydraulic calcium silicate cements are widely employed in endodontics due to their excellent biocompatibility, bioactivity, sealing ability, and remineralisation potential. However, currently used products such as mineral trioxide aggregate (MTA) exhibit several limitations, including discolouration, cost, and poor handling properties. Therefore, this study aimed to develop and characterise a bovine‐derived hydroxyapatite (BHA) and montmorillonite (MMT) nanoclay hybrid composite (BHA‐MMT), incorporating zirconium oxide as a radiopacifier and calcium silicate phases as setting agents.

**Methodology:**

BHA was produced from waste bovine bones using a defatting and deproteination method. MMT clay was exfoliated into nanoclay using shear force with a planetary ball mill. The composite powders were prepared by combining BHA (20% and 25% wt), zirconium oxide (30% wt), setting agents with exfoliated MMT (15% and 20% wt) to produce three composite materials with different BHA and MMT ratios, namely B20, B25, and M20. The resulting powders were mixed with deionised water to form hydraulic cements. The composite materials were tested for chemical, mechanical, and physical properties compared with the commercially available ProRoot MTA. The biocompatibility, bioactivity, and remineralisation potential of the materials were also examined using established in vitro *assays*.

**Results:**

The pH of the material increased above 11 after 24 h and remained constant up to 21 days. The compressive strengths of B20, B25, and M20 were 23.63, 21.2, and 23.8 MPa, respectively, after 21 days. The mean setting time of the composites ranged from 13 to 14 min, and the concentrations of heavy metals were within the permissible limits specified by ISO standards. The experimental composite materials demonstrated radiopacity values within the range, consistent with ISO recommendations. Among the variants, B20 and B25 demonstrated good cell viability, while M20, containing a higher proportion of MMT, showed enhanced apatite formation and remineralization, indicating improved bioactivity.

**Conclusion:**

The developed BHA–MMT composites demonstrated promising physicochemical, biological, and remineralisation properties consistent with ISO standard requirements. Overall, the product represents a bioactive composite material system; further investigations, including in vivo studies, are necessary to confirm its clinical applicability in vital pulp treatment (direct/indirect pulp capping, pulpotomy) and root‐end filling procedures.

## 1 Introduction

Caries is a common disease that if left untreated, can progress rapidly through the dentine, causing pulpitis and eventually pulp infection and necrosis. Pulpitis can be managed by vital pulp treatment techniques followed by restoration of the tooth. However, if the pulp bleeding cannot be controlled, or the pulp is necrotic, alternative treatment may be root canal treatment and restoration (Douglass and Douglass 2003). Vital pulp treatment and root canal treatment for teeth with open apices and apical microsurgery use restorative materials that are primarily bioceramic in nature are called hydraulic calcium silicate cements (HCSCs), typically formulated from calcium silicate, calcium phosphate, or bioactive glass. HCSCs, including mineral trioxide aggregate (MTA), are widely regarded as first‐line materials for root‐end filling procedures and vital pulp treatment techniques due to their biocompatibility, sealing ability, and capacity to promote hard tissue formation. However, despite their favourable biocompatibility and bioactivity, conventional MTA formulations and earlier generations of HCSCs have been associated with certain limitations, including high cost, potential tooth discolouration, prolonged setting time, and handling difficulties, although these features are not universal across newer HCSC formulations. Among these drawbacks, the high cost (Parirokh and Torabinejad 2010), tooth discolouration (Tripathi et al. 2020), long setting time (Parirokh and Torabinejad 2010), poor handling properties (Pushpalatha et al. 2022), and the presence of heavy metals are major concerns associated with conventional MTA (Camilleri et al. 2020; Chang et al. 2010).

Hydroxyapatite (HA) is a calcium phosphate mineral commonly present in bones and teeth. It forms direct chemical bonds with other living tissues due to its biocompatibility, non‐toxicity, non‐inflammatory, and non‐immunogenic nature (Sobczak et al. 2009). HA derived from natural sources, such as bovine bone, has shown superior physical and biological properties compared with synthetic hydroxyapatite due to its similarity to the mineral composition of natural bone and teeth (Ayatollahi et al. 2015; Ratnayake et al. 2017). However, it is challenging to incorporate hydroxyapatite into endodontic bioceramic materials due to its sub‐optimal mechanical properties (Bulut et al. 2016).

**FIGURE 1:**
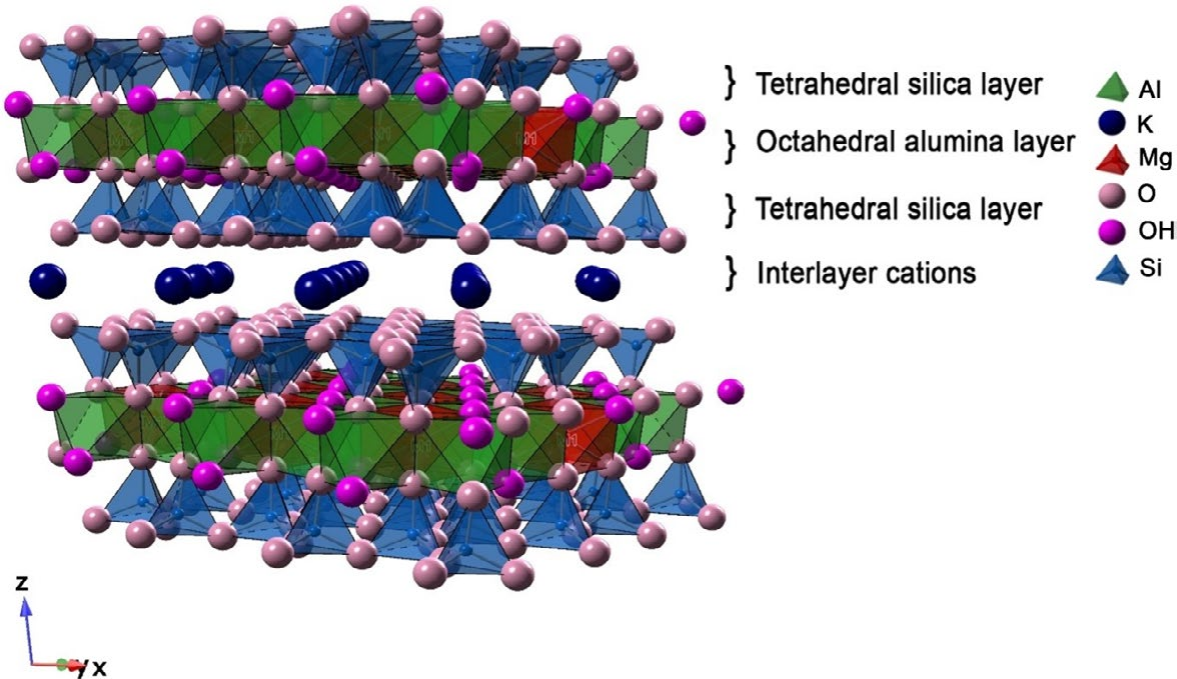
Crystal structure of montmorillonite showing its 2:1 layered arrangement, with an octahedral sheet (Al^3+^ partly substituted by Mg^2+^) sandwiched between two tetrahedral silica sheets, and interlayer spaces containing exchangeable cations and water molecules (CrystalMaker v10).

To address this limitation, montmorillonite (MMT), a nanoclay mineral, may offer a solution by providing effective reinforcing properties (Solhi et al. 2012; Dowling et al. 2006). MMT is a biocompatible, affordable phyllosilicate mineral clay (Figure 1) with a nanolayered structure. It has a high specific surface area and high cation exchange capacity. MMT is approved by the United States Food and Drug Administration (US‐FDA) for drug delivery applications, and recently, it has gained attention due to its ability to be used in prolonged‐release drug delivery systems (Inamuddin and Mohammad 2018). Several studies have reported the incorporation of exfoliated MMT nanoclay into composite materials to enhance their mechanical and physical properties (Wang et al. 2008; Arbelaiz et al. 2021; Peramune Arachchilage et al. 2024). MMT can be exfoliated for dental composite use through high‐shear mixing, ultrasonication, organo‐modification, solvent swelling, or in situ polymerisation. These techniques separate the stacked layers, which increases surface area and improves resin compatibility, with the most effective results often achieved by combining organo‐modification with mechanical dispersion (Zhu et al. 2019).

Recognising the reinforcing potential of montmorillonite (MMT) and the well‐established biocompatibility of hydroxyapatite, combining these materials may offer advantages in the development of bioactive endodontic materials suitable for vital pulp therapy and other endodontic applications. Therefore, the aim of this study was to develop and evaluate a bioactive endodontic composite by incorporating bovine‐derived hydroxyapatite (BHA) and montmorillonite (MMT) together with calcium silicate components. The hypothesis of this study was that the incorporation of BHA and MMT could enable the development of a bioactive endodontic material with adequate physicochemical properties and improved biocompatibility for endodontic applications. The material was designed for vital pulp treatment applications, including direct and indirect pulp capping, pulpotomy, root‐end filling, and perforation repair. In addition, the material was characterised in accordance with relevant ISO standards to assess its physicochemical properties and overall suitability for these endodontic applications. The composite was designed to provide fast‐setting behaviour, high bioactivity, and adequate compressive strength, making it well‐suited for pulp‐capping and related procedures in which biological performance is prioritised over high load‐bearing capacity.

## 2 Materials and Methods

The manuscript of this laboratory study was written according to the Preferred Reporting Items for Laboratory Studies in Endodontology 2021 guidelines (Nagendrababu et al. 2021), and the flowchart is summarised in Figure S1. Ethical approval was not required and not applicable for this study.

### 2.1 Preparation of BHA–MMT Hybrid Composite Material

The experimental formulation consisted of bovine‐derived hydroxyapatite (BHA) and MMT, with zirconium oxide added to provide radiopacity. Tricalcium silicate and dicalcium silicate served as the main hydraulic setting components, while calcium carbonate and calcium sulphate were included to regulate setting behaviour. The material was subsequently characterised and evaluated in accordance with relevant ISO quality parameters.

Bovine hydroxyapatite (BHA) was prepared using the method previously developed by Ratnayake et al. involving a defatting and deproteinisation technique (Ratnayake et al. 2017, 2020; Ramesh et al. 2020). The samples were ground using a mortar and pestle, followed by milling for 1 h at 400 rpm using a planetary ball mill (PQN2.110; Across International LLC, Livingston, New Jersey, USA). Raw montmorillonite K 10 (Sigma‐Aldrich, St. Louis, MO, USA) was ball‐milled at 400 rpm for 2 h to obtain exfoliated MMT nanoclay for use in the composite material (Chatterjee et al. 2017). Based on pilot studies and previous literature (Camilleri et al. 2011), Zirconium oxide powder, 5 μm, 99% trace metals basis (Sigma‐Aldrich, St. Louis, MO, USA) was incorporated at a fixed percentage of 30 wt%, which was maintained constantly across all composite material specimens. ZrO_2_ was milled at 300 rpm for 20 h with a ball‐to‐powder ratio of 10:1 to obtain uniform particle sizes. Uniform and reduced particle sizes further allow better packing of the material and yield a defect‐free, dense structure. Finally, all powders forming the hydraulic setting binder (tricalcium silicate and dicalcium silicate; Nanochemazone, Chemzone Inc., Leduc, Alberta, Canada), along with calcium carbonate and calcium sulphate (Sigma‐Aldrich, St. Louis, MO, USA) were combined in the ratios specified in Table 1 using a ball mill at 300 rpm for 1 h to obtain a uniform powder blend. The resulting powders were sintered at 850°C for 5 h in a muffle furnace. Following sintering, the powders were again ball milled for 1 h at 300 rpm to obtain the final powder mixture:

**TABLE 1:** Percentage components (% wt) of the BHA‐MMT‐based composite material.

Sample name	MMT	BHA	ZrO_2_	Tricalcium silicate	Dicalcium silicate	Calcium carbonate	Calcium sulphate
B20	15	20	30	17.5	5.25	10.5	1.75
B25	15	25	30	15.0	4.5	9.0	1.5
M20	20	20	30	15.0	4.5	9.0	1.5

### 2.2 Evaluation of Physical, Mechanical and Chemical Parameters

The specimens were prepared according to the dimensions specified by the respective standards. The powder blend was placed on a glass mixing slab, thoroughly mixed with 350 μL/g of distilled water using a dental cement spatula, and then introduced into the moulds as described below.

#### 2.2.1 Colour and Colour Stability

The colour stability of the BHA‐MMT composite materials was analyzed based on an adapted method (Camilleri 2014), with bovine blood replacing traditional hydrogen peroxide. BHA‐MMT composite materials were prepared as described in Section 2.2, using moulds with an internal diameter of 9 mm and a thickness of 2 mm. ProRoot MTA (Dentsply‐Sirona, Baillagues, Switzerland) was used as the reference material and prepared according to the manufacturer's (Dentsply‐Sirona) instructions. The samples were initially allowed to set for 24 h at 37°C under 100% humidity. After this period, the specimens were immersed in water, 2.5% sodium hypochlorite (Sigma‐Aldrich, USA), and locally sourced bovine blood. The samples were then stored at 37°C with 100% humidity and visually inspected after 24 h and 7 days for colour change.

#### 2.2.2 Radiopacity

BHA–MMT composite and ProRoot MTA specimens (*n* = 6) were fabricated using 3D‐printed moulds made of thermoplastic polyurethane (TPU) (Kiwi3D, Queenstown, New Zealand) with an internal diameter of 10 mm and a thickness of 1 mm, in accordance with ISO 6876 (Standardization and Standardization 2001). The specimen disks were placed on size 2 photostimulable phosphor plates (PSP‐2; Dentsply Sirona, Germany), adjacent to an aluminium stepwedge with 1 mm step increments (99.5% pure, AFP Manufacturing, USA), in compliance with ISO 6876:2012. Radiographic exposure was performed using a dental X‐ray unit (Dentsply Sirona, NZ) operating at 60 kV, 7 mA, and 0.32 s. The digitised images were processed using a Dentsply Xios PSP Scanner (Dentsply Sirona, NZ) and Sidexis imaging software (Dentsply‐Sirona, NZ) and exported as JPEG files. A calibration curve was generated using ImageJ software (National Institutes of Health, Bethesda, MD, USA), converting the radiographic density values to aluminium stepwedge equivalent thicknesses. The radiographic density of each composite sample was reported as the average value obtained from six radiographic measurements (*n* = 6). Radiopacity data were analysed using one‐way analysis of variance (ANOVA), with values expressed as mean ± standard deviation.

#### 2.2.3 Setting Time

The setting time was determined according to ISO 6876 (Standardization and Standardization 2001), which specifies procedures for endodontic materials requiring moisture to set. A gypsum mould was prepared by placing freshly mixed gypsum (USG Ultracal 30, USG, Illinois, USA) on a Petri dish. Plastic disks (10 mm in diameter × 1 mm in thickness) were then pressed into the unset gypsum surface to create cavities with the dimensions specified. The gypsum moulds were then placed in an incubator (Labec, Marrickville, NSW, Australia) for 24 h at 37°C in 100% humidity prior to testing. From each group, three samples (*n* = 3) were placed into the disk‐shaped cavities of the gypsum mould immediately after mixing. A Vicat apparatus (Humboldt Mfg, Elgin, IL, USA) with a flat‐ended 1.0 mm needle and a 300 g weight was used for the setting time analysis (Zarra et al. 2018; Abu Zeid and Edrees 2022). The Vicat apparatus needle was lowered vertically onto the surface of the setting material, then raised to check for an indentation. The process was repeated until no visible indentation was observed. Time measurements for indentation were taken initially every 5 min and then every minute thereafter until signs of setting were observed (Yong et al. 2022).

#### 2.2.4 Compressive Strength

Compressive strength of the experimental samples (*n* = 10 per group) was determined by the method described in ISO 9917‐1 using specimens with dimensions of 4 mm × 6 mm (Standardization 2007). The specimens were prepared after mixing the powder blend as per the ratio (Table 1) with deionised water and allowed to set for 24 h. The compressive strength was measured along the long axis of the samples as specified in the ISO standards using an Instron 3369 (Instron, Boston, Massachusetts, USA). The effect on compressive strength over time was evaluated by placing the samples on moistened paper towels and incubating them at 37°C with 95% relative humidity (Labec, Marrickville, NSW 2204, Australia) for 21 days. The data were analysed using ANOVA with Tukey's post hoc test, with values expressed as mean ± standard deviation.

#### 2.2.5 X‐Ray Diffraction Analysis

X‐ray diffraction (XRD) analysis was performed after hydration and setting to detect phases such as calcium silicate hydrates and hydroxyapatite in the BHA‐MMT composite material and to assess the material's stability, hydration, and bioactivity. The analysis was carried out using a PANalytical Empyrean X‐ray diffractometer (Malvern Panalytical Ltd., Malvern, UK) in θ–θ configuration with a reflection–transmission stage and spinner sample holder. A Cu anode source (*Kα*_1_ = 1.5406 Å, *Kα*2 = 1.5444 Å, intensity ratio = 0.5) was operated at 45 kV and 40 mA. Data were collected without a monochromator, using a fixed 0.5° divergence slit. Scans were performed in continuous mode over a 2*θ* range of 0°–50°, with a step size of 0.02626°, yielding 1904 data points. Diffraction patterns (intensity vs. angle) were obtained using the PANalytical software for phase identification and further analysis.

#### 2.2.6 Fourier Transform Infrared Analysis

Fourier Transform Infrared (FT‐IR) analysis was performed to identify the functional groups and chemical bonds present in the BHA‐MMT composite, confirming hydration reactions, phase composition, and the formation of apatite. FT‐IR spectra were recorded using a Bruker ALPHA II spectrometer (Bruker Optics GmbH & Co. KG, Ettlingen, Germany) (equipped with a diamond ATR module). BHA‐MMT composite samples were placed directly on the crystal, and spectra were collected in the 4000–400 cm^−1^ range with a resolution of 4 cm^−1^, averaging 64 scans per measurement. Background spectra were acquired prior to each run, and the ATR crystal was cleaned with ethanol between samples. The obtained spectra were baseline‐corrected, ATR‐compensated, and analysed using OPUS software for functional group identification.

#### 2.2.7 ICP‐MS Elemental Analysis

The BHA‐MMT composites and ProRoot MTA (Dentsply‐Sirona) specimens were digested at room temperature overnight in freshly prepared *aqua regia* (3:1 HCl:HNO_3_). Elemental analysis was conducted using an Agilent 7900 inductively coupled plasma mass spectrometer (ICP‐MS) (Agilent Technologies, Waldbronn, Germany). Elemental concentrations were determined from internal standard‐corrected external calibration curves to ensure high sensitivity and analytical precision. All measurements were performed in duplicate, and results are reported as mean ± standard deviation.

#### 2.2.8 Solubility

The solubility of the BHA–MMT composites was evaluated according to ISO 6876 (Standardization and Standardization 2001). Specimen disks (20 mm in diameter and 1.5 mm in height) were fabricated using a mould and weighed to the nearest 0.001 g. Two disks were placed in a 90 mm Petri dish containing 50 mL of deionised water and stored at 37°C for 24 h. The water was then filtered through fluted filter paper (Whatman Grade 1, UK), and the filtrate was evaporated in an oven at 110°C. The weight of the residual solid was recorded using a precision balance (Voyager Pro Balance, Ohaus, NJ, USA), and solubility was calculated to the nearest 0.1% from the difference between the initial specimen weight and the weight of the dissolved matter. Triplicate readings were obtained by repeating the procedure three times (*n* = 3).

#### 2.2.9 Effect of the BHA‐MMT Materials on pH

The pH of the BHA‐MMT composites was determined using the method previously described (Poggio et al. 2017). The composite material specimens were synthesised as 2 mm in thickness and 10 mm in diameter disks using a TPU (Kiwi3D) mould (Poggio et al. 2017). The specimen disks were freshly made and placed in separate glass vials containing 10 mL of distilled water. The specimens were incubated at 37°C with 95% humidity (Labec, Marrickville NSW 2204, Australia) for 3 h before the first pH measurement. The pH measurements of the distilled water were obtained using a temperature‐calibrated pH probe (InLab 413 IP67, Mettler‐Toledo, Greifensee, Switzerland) at 3 h, 24 h, 7 days, and 14 days.

### 2.3 In Vitro Biocompatibility

The biocompatibility of the BHA‐MMT composite materials was evaluated using the Saos‐2 osteosarcoma cell line (ATCC HTB‐85, USA) with the LIVE/DEAD cell viability/cytotoxicity assay (Invitrogen Life Technologies, Carlsbad, CA, USA) and the MTS assay (CellTiter 96 Aqueous One Solution, Promega, WI, USA).

#### 2.3.1 Composite Material Preparation for Cell Culture

Composite material disks and ProRoot MTA measuring 8 mm in internal diameter and 2 mm in height were fabricated and allowed to set overnight at 37°C under 100% humidity. The samples were then surface‐sterilised using UV light (200–280 nm) for 15 min and immersed in 70% ethanol under UV light in a biosafety cabinet for 30 min. Afterwards, the samples were rinsed three times with sterile phosphate‐buffered saline (PBS) to remove residual ethanol, immersed in 1 mL of culture medium, and equilibrated for 24 h with medium replaced every 24 h.

#### 2.3.2 Cell Culture

Saos‐2 cells were cultured for both live/dead cell viability assay and cell proliferation assay in a T25 *Standard Cell Culture Flask T‐25 (CELLSTAR, Germany) at* 37°C in a humidified atmosphere containing 5% CO_2_. Cultures were grown in Minimum Essential Medium (α‐MEM) (ThermoFisher Scientific, Auckland, New Zealand) supplemented with 10% foetal bovine serum (FBS) (ThermoFisher Scientific, Auckland, New Zealand) and 1% antibiotic‐antimycotic solution (ThermoFisher Scientific, Auckland, New Zealand) (Yong et al. 2022) with media changes as follows.

#### 2.3.3 LIVE/DEAD Cell Viability/Cytotoxicity Assay

Live/Dead cell viability assay was performed following the protocol described by Yong et al. (2022). Saos‐2 cells (6000 cells per disk) were seeded onto the surface of each disk in a 48‐well plate (Thermo Scientific *Nunc*, Denmark). The disks were incubated for 1 h to allow cell attachment, after which time 1 mL of culture medium was added. Samples were then incubated at 37°C in a humidified atmosphere containing 5% CO^2^ for 24 and 72 h. The culture medium was replaced daily throughout the experimental period. At each time point, the culture medium was removed, the samples were washed with PBS, stained with Live/Dead Viability/Cytotoxicity Kit (Thermo Fisher Scientific, USA), and observed under a fluorescence microscope (Invitrogen EVOS M5000 Imaging System, Thermo Fisher Scientific, MA, USA). Live cells were visualised as green fluorescence, whilst dead cells appeared red. The percentage of live cells was determined using the manufacturer's EVOS software (Thermo Fisher Scientific, MA, USA). Images were captured from three random fields per sample for qualitative analysis.

#### 2.3.4 Cell Proliferation Assay

Cell proliferation of the BHA–MMT composite materials was determined using the MTS assay, a colourimetric method in which the colour change is directly proportional to the number of viable cells. A standard curve (40000–3750 Saos‐2 cells) was produced for each trial. Cell proliferation was quantified using a microplate reader (CLARIOstar Plus, BMG LABTECH GmbH, Ortenberg, Germany) at 490 nm. The absorbance values obtained were then correlated with the standard curve to calculate the cell number. Assays were conducted at 3 time points: 24, 48, and 72 h. A two‐way analysis of variance (ANOVA) was used to assess the effects of material type and incubation time (24, 48, and 72 h) and their interaction on cell proliferation. Post hoc multiple comparisons were performed using Tukey's test.

#### 2.3.5 Apatite‐Forming Potential

Four samples of B20, B25, M20, and the reference material ProRoot MTA were placed into the wells of a 48 well plate and covered with Dulbecco's PBS (DPBS). The samples were incubated at 37°C with 100% humidity for 7 days. After this period, apatite formation on the samples was observed using SEM and EDX spectroscopy (Zeiss Sigma VP FEG SEM; Zeiss, Tokyo, Japan) (Gandolfi et al. 2011).

#### 2.3.6 Alizarin Red Staining

The composite and ProRoot MTA disks were fabricated as described earlier. Saos‐2 cells (8000 cells) were cultured in the presence of sample disks using osteogenic medium containing 10 mmol/L β‐glycerophosphate, 50 μg/mL L‐ascorbic acid, and 100 nmol/L dexamethasone (Rafatjou et al. 2018). The control group was also cultured without sample disks. Osteogenic medium was replaced every 3 days for 21 days. Then the composite and ProRoot MTA disks and osteogenic medium were removed. The cell‐well plate construct was washed with DPBS, fixed with 10% formaldehyde, and then stained with alizarin red solution for 20 min at room temperature. Digital images were captured using a fluorescence microscope (Invitrogen EVOS M5000 Imaging System, Thermo Fisher Scientific, USA). For quantification, 400 μL of 10% acetic acid was added to each well and incubated for 30 min. Cells were scraped, collected into microcentrifuge tubes, vortexed, heated at 85°C for 10 min, cooled on ice, and centrifuged at 20 000 × *g* for 15 min. The supernatant (400 μL) was neutralised with 150 μL of 10% ammonium hydroxide, and 150 μL of each sample was transferred to a 96‐well plate for absorbance measurement using a microplate reader (CLARIOstar Plus, BMG LABTECH GmbH, Ortenberg, Germany) at 405 nm. Triplicate readings were analysed using ANOVA with Tukey's post hoc test, with values expressed as mean ± standard deviation.

### 2.4 Statistical Analysis

Statistical analysis was carried out using IBM SPSS Statistics version 25. Data were presented as mean ± standard deviation (SD). Differences between groups were first analysed using one‐way ANOVA, and Tukey's post hoc test was applied when pairwise comparisons were needed. A two‐way ANOVA was performed to compare cell proliferation among the material variants and across different incubation time points. A *p*‐value below 0.05 was considered statistically significant.

## 3 Results

### 3.1 Physical Characterisation

#### 3.1.1 Colour and Colour Stability

**FIGURE 2:**
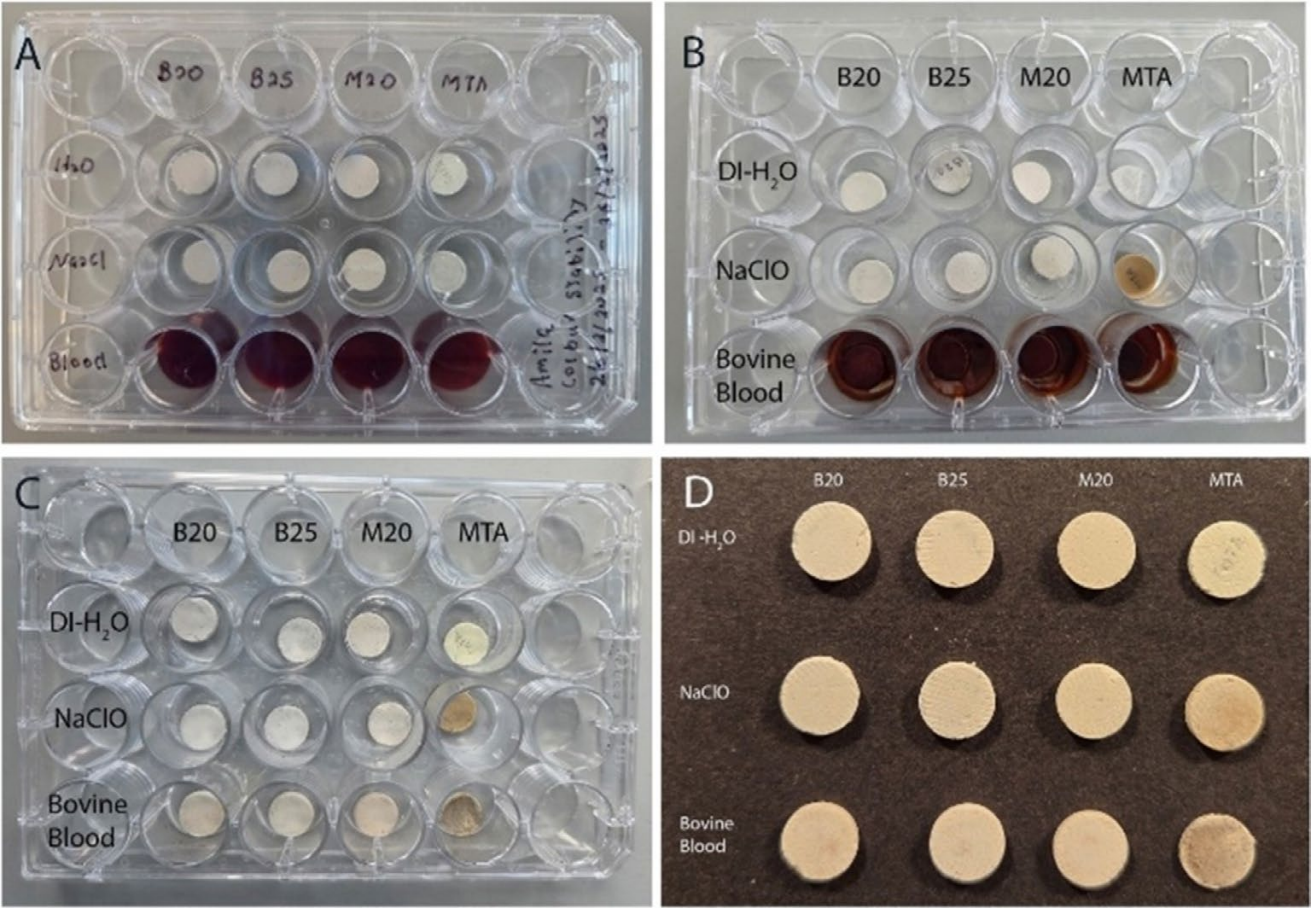
Representative samples showing the colour change of the experimental composite materials and ProRoot MTA in deionised water, sodium hypochlorite (NaOCl) (2.5%), and bovine blood at different stages: (A) original colour before immersion, (B) after 24 h of immersion, (C) after the removal of water, sodium hypochlorite and bovine blood, (D) after drying the samples overnight.

The colour stability of the BHA‐MMT composites once fully set appeared pale white, whereas ProRoot MTA was pale yellow (Figure 2 and Figure S2). Qualitative analysis showed that all three BHA‐MMT composite samples maintained their colour in water, sodium hypochlorite, and bovine blood. In contrast, ProRoot MTA turned light brown in sodium hypochlorite and grey in bovine blood (Figure 2), demonstrating that the BHA‐MMT composites remained stable in 2.5% sodium hypochlorite, a widely used gold‐standard irrigant for root canal treatment (Duncan et al. 2023).

#### 3.1.2 Radiopacity

**FIGURE 3:**
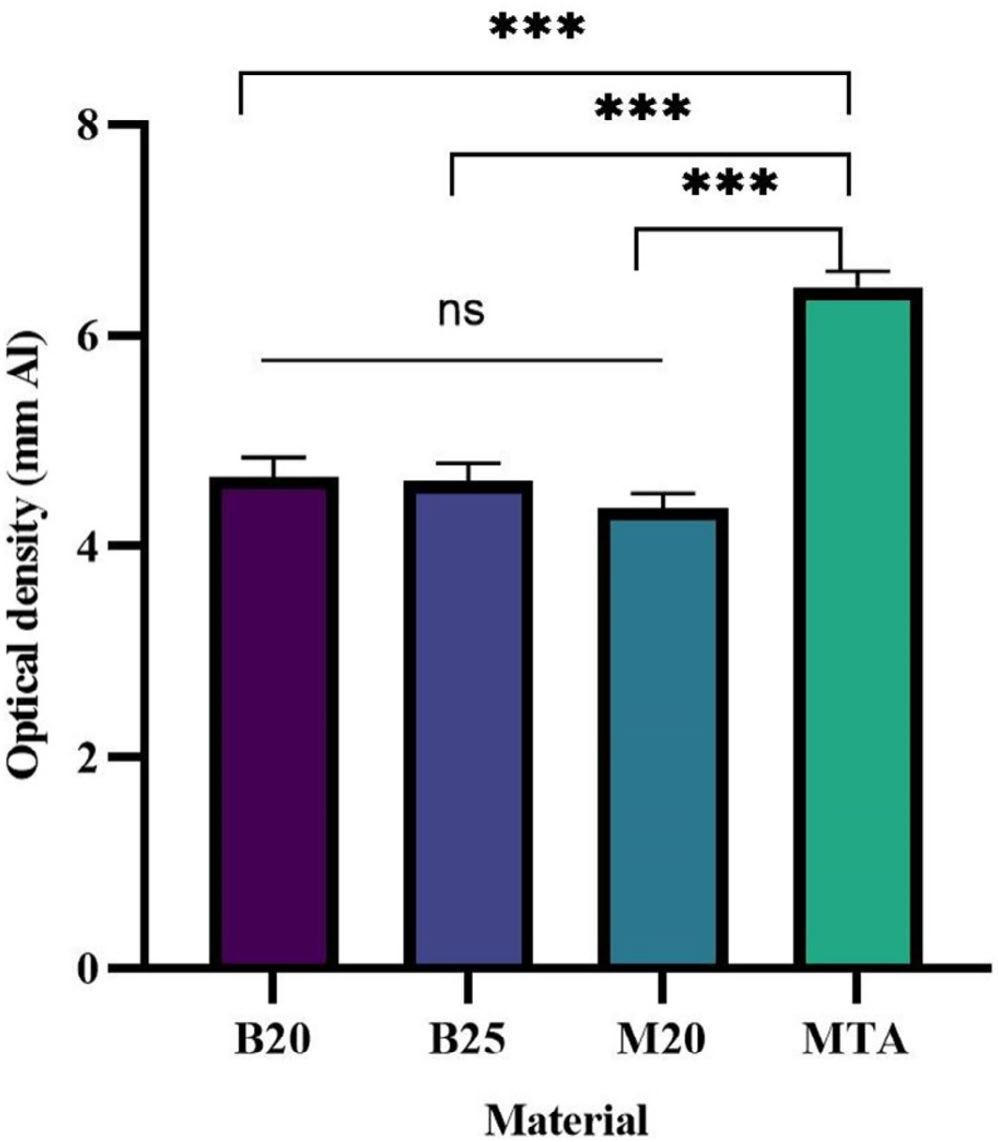
Radiopacity (Optical density) values of the BHA–MMT composite samples and ProRoot MTA. Error bars represent the standard deviation of the mean (*n* = 6) after one‐way ANOVA with Tukey's post hoc test; ns = not significant; ***p < 0.001.

Analysis of the composite samples indicated adequate radiopacity levels for clinical application and was compliant with ISO 6876:2012 (minimum of 3 mm Al). The radiopacity of each composite is shown in Figure 3. The B20 sample showed the highest optical density value. ProRoot MTA (Dentsply‐Sirona) showed an optical density value of 6.46 ± 0.37 mm Al. Statistically, the MTA samples had a significantly higher mean optical density value (*p* < 0.05), while no significant differences were observed among the BHA‐MMT composite samples (*p* > 0.05).

#### 3.1.3 Compressive Strength

The compressive strength values of the BHA‐MMT and ProRoot MTA after 24 h and 21 days are shown in Table 2. All materials showed a statistically significant increase in strength over time (*p* < 0.05), with the experimental groups B20, B25, and M20 showing a 13.6%, 10.4%, and 15.0% increase, respectively. Moreover, ProRoot MTA demonstrated a significantly higher compressive strength at both time points compared with the experimental groups, increasing from 41.3 ± 1.49 MPa to 70.73 ± 1.70 MPa (*p* < 0.05).

**TABLE 2:** Compressive strength of BHA‐MMT composite materials and ProRoot MTA (*n* = 10).

	Compressive strength (MPa)	
	24 h	21 days
B20	20.8 ± 0.91	23.63 ± 2.32
B25	19.2 ± 1.31	21.2 ± 2.59
M20	20.7 ± 1.04	23.81 ± 4.01
ProRoot MTA	41.3 ± 1.49	70.73 ± 1.7

#### 3.1.4 Setting Time

**FIGURE 4:**
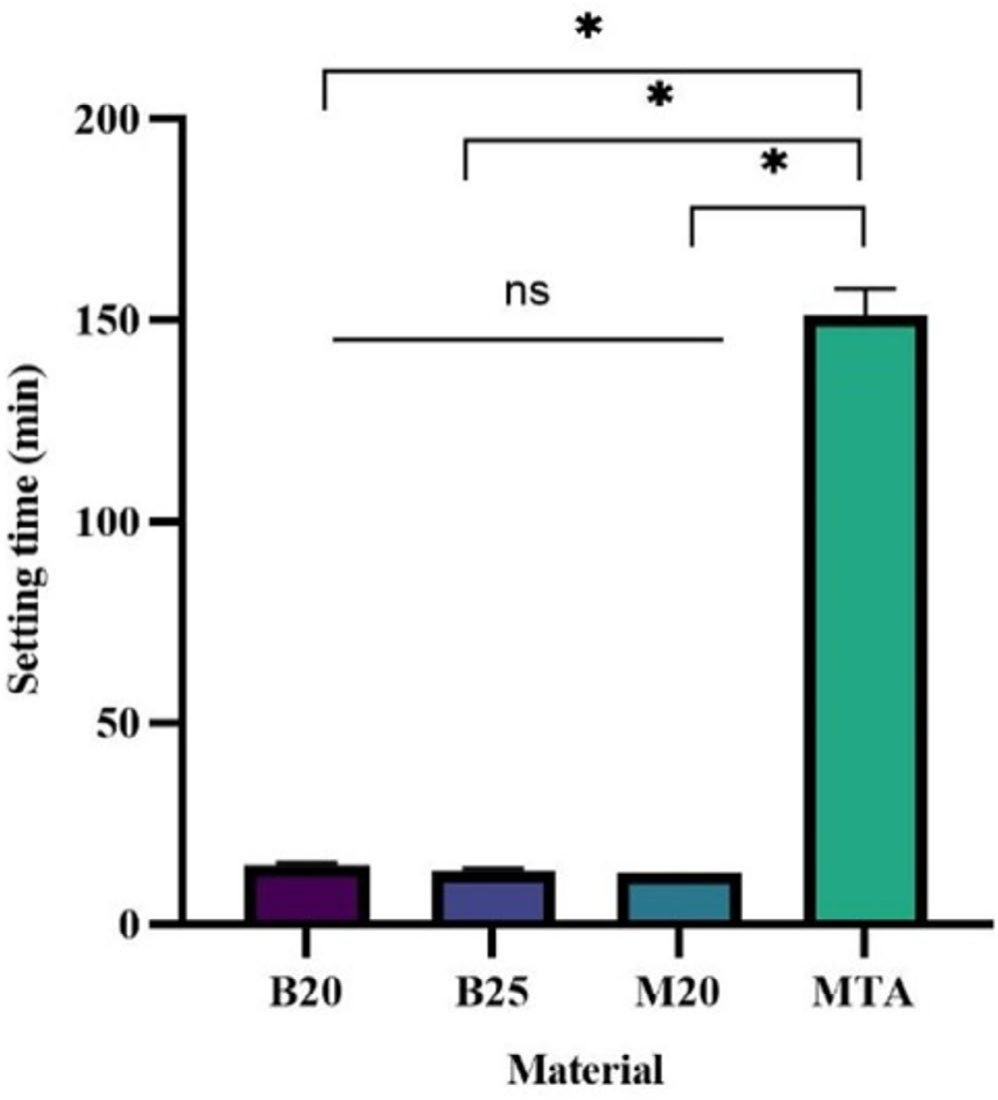
Setting time of the three composite materials. Error bars represent the standard deviation (SD) (*n* = 3). Statistical analysis—one‐way ANOVA with Tukey's post hoc test; *p < 0.05.

The setting time results showed that all BHA–MMT composites set within 12–15 min. No statistically significant differences were observed among B20, B25, and M20. In contrast, ProRoot MTA exhibited a significantly longer setting time compared with the BHA–MMT composites (*p* < 0.05). The mean setting times were 14, 13, and 13 min for B20, B25, and M20, respectively (Figure 4).

#### 3.1.5 ICP‐MS Elemental Analysis

Based on ICP‐MS elemental analysis (Table 3), the BHA‐MMT composite material is primarily composed of Ca and P as the main elemental constituents. Additionally, Fe, Mg, Na, and K were also detected in the samples. Furthermore, the data indicated the presence of heavy metals such as Pb, Cr, Cd, As, and Bi, all of which were well within the permissible limits specified by ISO standards and almost identical levels or lower than those in ProRoot MTA according to the analysis (Standardization and Standardization 2001).

**TABLE 3:** Acid‐soluble content of B25 composite and ProRoot MTA cement determined by ICP‐MS after overnight setting.

Element	B20 Weight of element (mg/kg)	B25 Weight of element (mg/kg)	M20 weight of element (mg/kg)	ProRoot MTA Weight of element (mg/kg)	Detection limit per 50 mg sample	Maximum Limit (ISO 9917‐1 2007)
Pb	1	1	1	< 0.5	0.5	100
Bi	< 20	< 20	< 20	< 20	20	
Ca	199 880	174 500	156 093	371 000	500	
P	60 890	40 750	34 988	685	500	
K	489	528	997	591	400	
Fe	1201	1210	1421	1685	10	
Mg	1987	2185	2987	2785	10	
Na	675	681	756	1110	10	
Ba	121	103	113	97	0.2	
Zn	39	33	45	7	5.0	
Cr	6	6	5	10	1.5	
Ni	1	1	1	44	0.5	
Cd	< 0.5	< 0.5	< 0.5	< 0.5	0.5	2
As	1	1	1	1 ± 0.03	0.5	

#### 3.1.6 FT‐IR Analysis of the Composite Material

**FIGURE 5:**
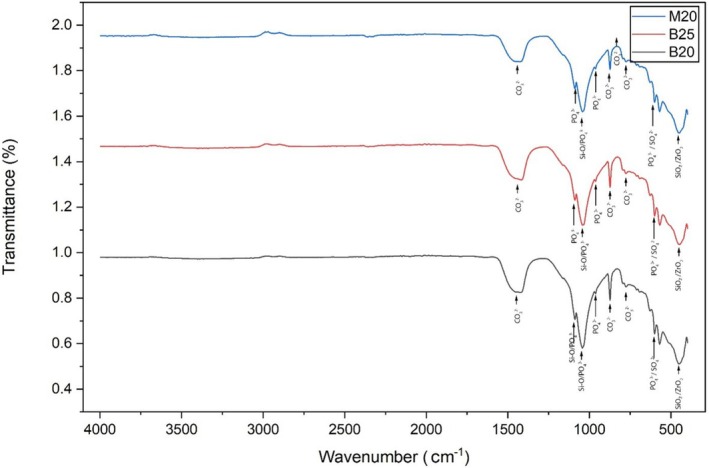
FT‐IR spectra of the BHA–MMT composites after heat treatment to 850°C.

FTIR spectra (Figure 5) revealed a dominant P‐O antisymmetric stretching vibration (${\mathrm{PO}}_{4}^{3-}$) at 1080 cm^−1^, together with a band at 960 cm^−1^ corresponding to the ${\mathrm{PO}}_{4}^{3-}$ symmetric stretch, both of which are characteristic of hydroxyapatite. The distinct bands at 1450 and 1421 cm^−1^ suggest asymmetric stretching of CO_3_^2−^, while the 874 cm^−1^ band indicates out‐of‐plane bending of CO_3_^2−^. The bands at 775 and 713 cm^−1^ correspond with the in‐plane bending modes of the CO_3_^2−^ group, indicating carbonate substitution or residual carbonate. The absorption bands observed at 600 and 560 cm^−1^ correspond to the ${\mathrm{PO}}_{4}^{3-}$ bending modes, with possible overlap from ${\mathrm{SO}}_{4}^{2-}$ groups in this region. Additionally, a band near 450 cm^−1^ was identified, which may arise from both silicate (Si–O–Si vibrations, attributed to montmorillonite residues) and zirconia (Zr–O–Zr bending), suggesting contributions from both phases in the composite (Figure 5).

#### 3.1.7 X‐Ray Diffraction Analysis of the Composite Material

**FIGURE 6:**
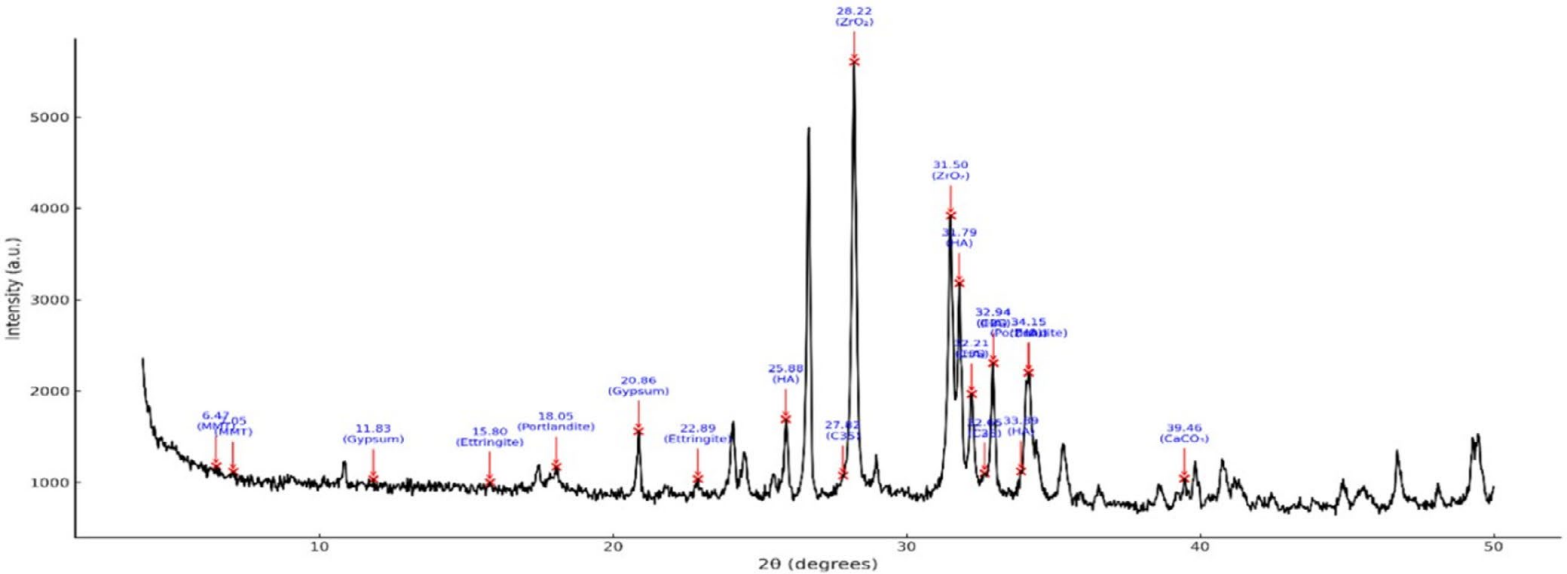
X‐ray diffraction pattern of the B25 composite.

X‐ray diffraction (XRD) data (Figure 6) confirmed that the composite material (B25) was composed of hydroxyapatite (HA), tricalcium silicate (C3S), dicalcium silicate (C2S), CaCO_3_, CaSO_4_, zirconium oxide (ZrO_2_), and montmorillonite. The presence of HA was confirmed with the major peaks at 25.8°, 31.7°, 32.2°, 32.9°, and 34.0° 2θ. The presence of tricalcium silicate (C3S) and dicalcium silicate (C2S) was confirmed with the characteristic reflections at 27.8°, 32.0°, 32.4°, and 32.8°. Calcite appears to be present, which may be associated with calcium silicate and calcium hydroxide hydration products as indicated by the reflections at 29.4°, 36.0°, and 39.5°. Characteristic reflections of montmorillonite are shown at 6.5°, 7.1°, and 19.8°, consistent with its layered silicate structure.

#### 3.1.8 pH and Solubility

The pH change of the BHA‐MMT composites and ProRoot MTA samples indicated that ProRoot MTA exhibited a higher pH after setting (Table 4). Out of all the samples, ProRoot MTA showed the highest pH values with a sharp increase in the first 24 h, followed by a gradual but slight decrease in pH while maintaining significantly higher pH compared with the other groups (*p* < 0.05). The experimental groups, especially B20, showed a moderately high initial pH after 3 h compared with B25 and M20 but did not show any significant differences statistically (*p* > 0.05). All three experimental groups showed an increase in pH up to Day 7 and remained constant until Day 21.

**TABLE 4:** pH measurement over time from 3 h to 21 days for the experimental composite material and MTA (*n* = 3).

Material	3 h	24 h	7 days	14 days	21 days
B20	10.19 ± 0.03	11.74 ± 0.03	11.80 ± 0.02	11.66 ± 0.01	11.61 ± 0.01
B25	9.88 ± 0.07	11.81 ± 0.02	11.90 ± 0.01	11.80 ± 0.04	11.74 ± 0.05
M20	9.49 ± 0.08	11.73 ± 0.04	11.75 ± 0.03	11.68 ± 0.04	11.64 ± 0.05
MTA	10.27 ± 0.08	11.88 ± 0.03	12.05 ± 0.07	12.06 ± 0.05	11.99 ± 0.01

The solubility assessment based on ISO standards (Table 5), B25 composite material recorded the highest mean solubility (1.89 ± 0.28), followed by B20 (1.51 ± 0.14) and M20 (1.09 ± 0.05). However, these differences were not statistically significant (*p* > 0.05).

**TABLE 5:** Mean solubility values of the novel BHA‐MMT composite materials (*n* = 3).

Composite	Mean solubility
B20	1.51 ± 0.16
B25	1.89 ± 0.75
M20	1.09 ± 0.69

### 3.2 Biocompatibility and Remineralisation

#### 3.2.1 LIVE/DEAD Cell Viability Assay

**FIGURE 7:**
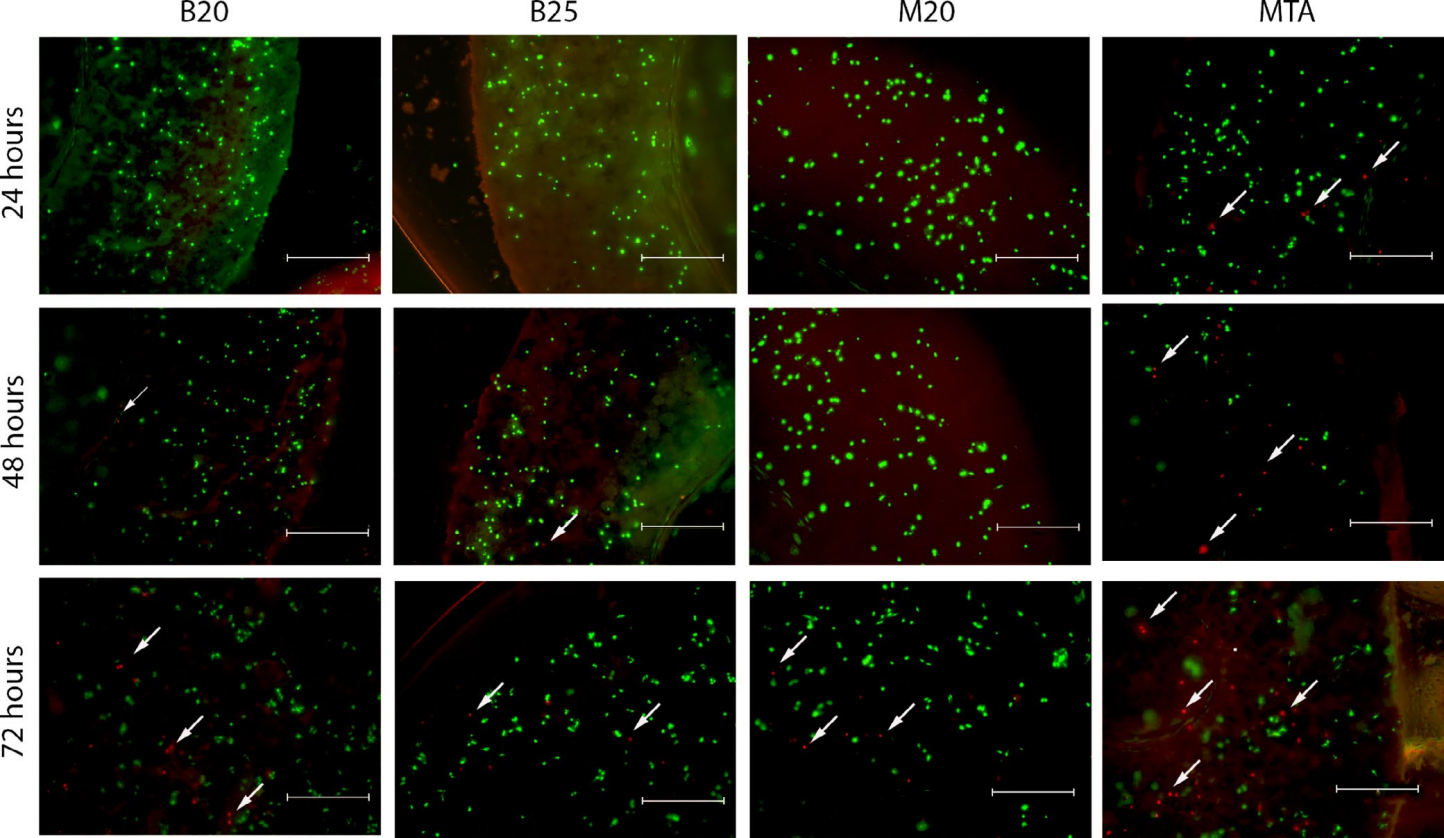
LIVE/DEAD Cell Viability Assay, representative fluorescent images showing Saos‐2 cells cultured over 72 h period on the BHA‐MMT composite and MTA (*n* = 3). Green = live cells (calcein), Red = dead cells (ethidium homodimer‐1). Scale bar = 750 μm.

Figure 7 shows the fluorescence images of the Live/Dead cell viability assay of the BHA‐MMT composite samples along with ProRoot MTA after 24, 48, and 72 h. All materials demonstrated excellent cell viability over the period from 24 to 72 h. A small number of dead cells were observed on the composite materials compared with the ProRoot MTA (arrows).

#### 3.2.2 Cell Proliferation Assay

**FIGURE 8:**
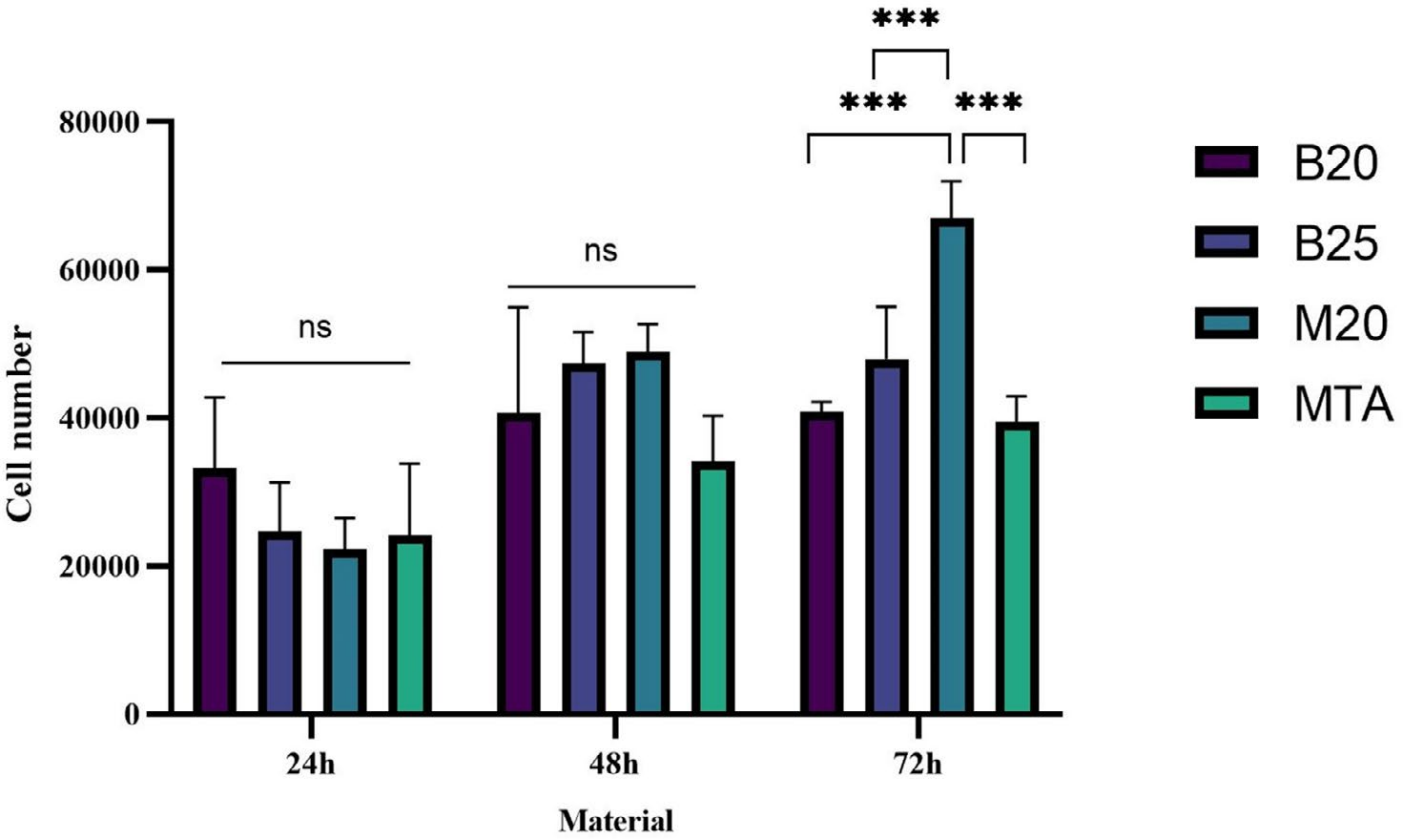
Cell proliferation analysis of Saos‐2 cells after seeding onto BHA‐MMT composites and MTA. Error bars represent the standard deviation (SD) (*n* = 3). (*n* = 3) after two‐way ANOVA with Tukey's post hoc test; ns = not significant; ***p < 0.001.

The MTS cell proliferation assay (Figure 8) showed an increase in cell number for all the tested materials from 24 to 72 h. The cell numbers increased significantly from 24 to 72 h; however, there was no significant difference between 48 and 72 h. Although there was no significant difference in cell numbers between the groups at 24 and 48 h periods, M20 had higher cell numbers than B20, B25, and MTA at 24 and 48 h. At 72 h, M20 had a significant increase in cell numbers compared with B20, B25, and MTA (all *p* ≤ 0.001).

#### 3.2.3 Alizarin Red Assay

**FIGURE 9:**
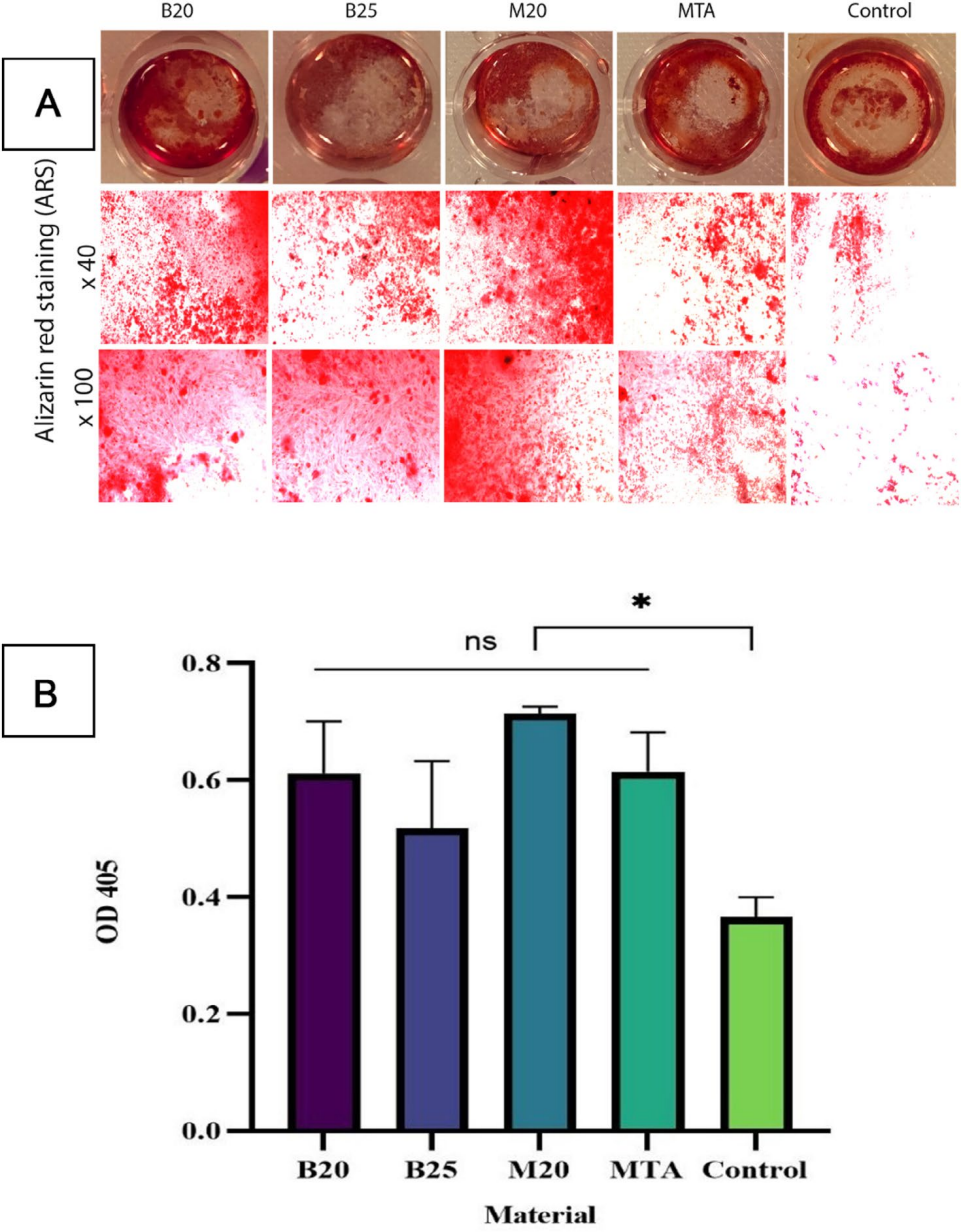
(A) Alizarin reds staining of the B20, B25, M20 composites, ProRoot MTA and control. (B) Quantitative analysis of mineral deposition is presented as OD 405 values for B20, B25, and M20 composites. Error bars represent the standard deviation (*n* = 3). Statistical analysis was performed using one‐way ANOVA with Tukey's post hoc test; ns = not significant, *p < 0.05.

Remineralisation of the experimental composite materials and ProRoot MTA was analysed using alizarin red staining (Figure 9) and by quantitatively comparing the absorbance values of the alizarin red staining (OD405). A control group was included in the assay, using Saos‐2 cells without exposure to any material.

**FIGURE 10:**
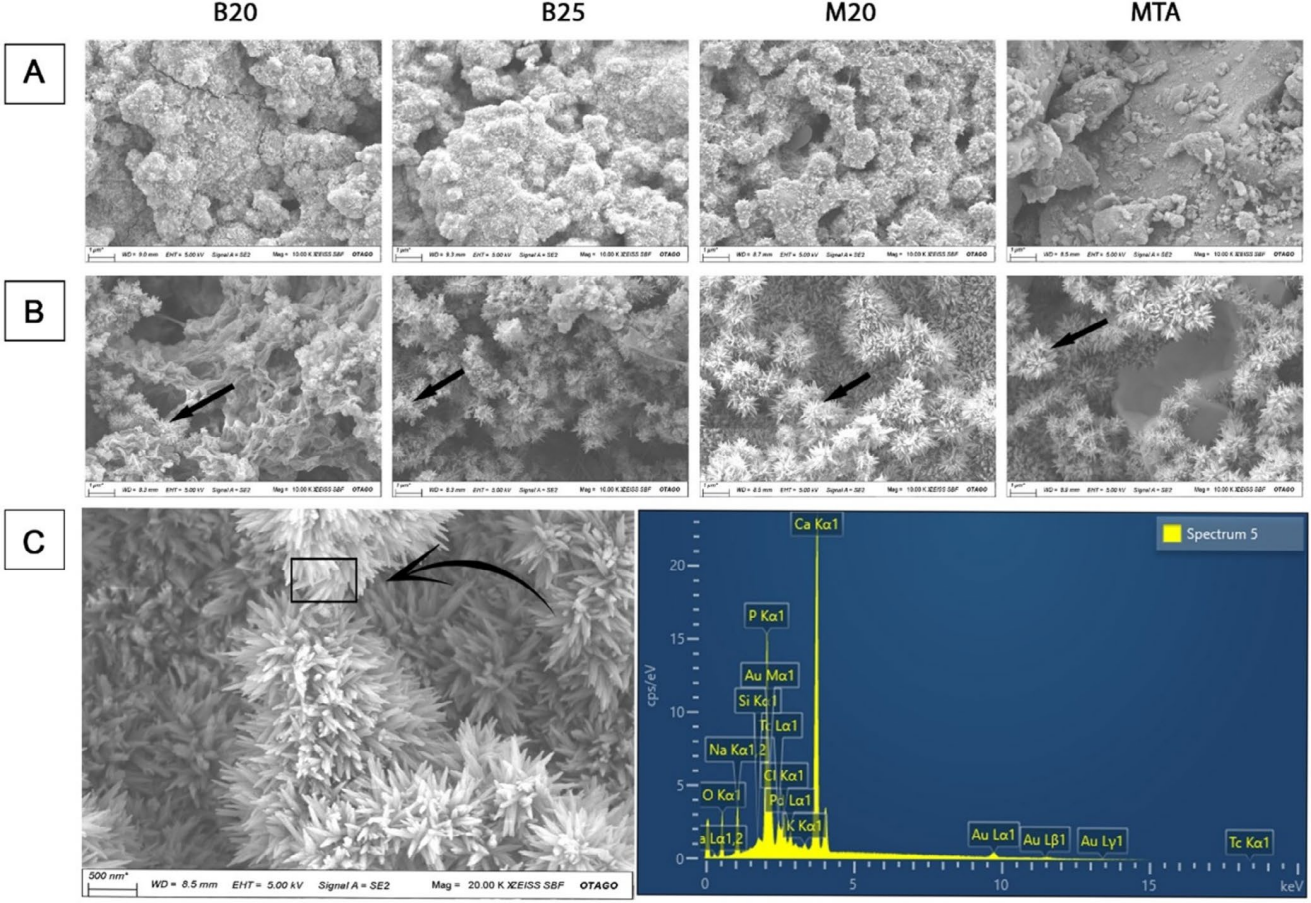
SEM images of BHA–MMT composites and ProRoot MTA (A) before immersion and (B) after immersion in DPBS for 7 days (10 000× magnification). (C) Area of interest (20 000× magnification) and corresponding EDS spectrum showing the crystalline deposits formed after immersion.

Alizarin red staining resulted in all groups exhibiting positive staining, indicating mineralised matrix formation, with variable intensities among the samples. Quantitative analysis demonstrated that the M20 composite showed the highest calcium deposition (Figure 10). However, there was no statistical difference among the tested composite groups and MTA (one‐way ANOVA; *p* > 0.05).

#### 3.2.4 Apatite Forming Potential

Apatite formed in varying degrees on each material surface after 7 days, with the deposition completely covering the M20 specimen (Figure 10). EDX spectral data revealed that the main constituents comprised calcium and phosphate with a Ca/P ratio of approximately 1.67, confirming hydroxyapatite formation.

## 4 Discussion

The aim of this study was to develop and evaluate a hybrid composite material based on bovine‐derived hydroxyapatite (BHA) and montmorillonite (MMT) clay for endodontic applications. The findings demonstrated that the developed composite exhibited favourable physicochemical characteristics and promising biological properties relevant to vital pulp treatment procedures. Notably, the material showed improved biocompatibility compared with MTA, while maintaining comparable physicochemical and mechanical properties, including adequate compressive strength for vital pulp treatment applications. Previous studies have shown that such bioactive phases promote mineralised tissue formation, dentine bridge development, and favourable pulpal/periapical outcomes (Posada‐Lotero et al. 2025). However, the incorporation of hydroxyapatite into endodontic materials is often limited by its relatively low mechanical strength (Bulut et al. 2016). In this formulation, MMT was therefore incorporated to improve handling and provide additional structural reinforcement, rather than to achieve high load‐bearing capacity.

Enhanced bioactivity and remineralisation are widely recognised as critical factors for successful outcomes in vital pulp treatment and regenerative endodontic treatment procedures. Experimental pulp healing models have demonstrated that materials capable of stimulating mineralised tissue formation and biological sealing support favourable pulpal responses and dentine bridge formation (Santos et al. 2021). In addition, clinical and translational studies have highlighted the importance of biologically active materials in promoting tissue regeneration and long‐term healing outcomes in endodontic applications (Meschi et al. 2023). In this context, the emphasis on bioactivity and remineralisation in the present composite material aligns with the biological requirements for pulp healing and effective apical sealing. Accordingly, the formulation was designed around conventional hydraulic calcium silicate chemistry, using tricalcium silicate and dicalcium silicate as the primary setting phases in combination with calcium carbonate and calcium sulphate.

Preliminary results indicated that this phase combination alone did not result in a stable set material and instead disintegrated upon immersion in water. A sintering step (850°C) was introduced to address this limitation, which significantly improved the setting and mechanical properties of the composite. The increased mechanical properties observed after sintering could be attributed to densification and stronger bond formation within the composite, which reduced porosity and reinforced the connections between BHA, MMT, and silicate particles (Prakasam et al. 2015; Nawang et al. 2019).

The physical characteristics of the material were assessed using standard protocols (ISO 6876; ISO 9917‐1) to determine its suitability for endodontic applications. The colour of the material after setting appeared white with a pale greyish tint, whereas ProRoot MTA (Dentsply‐Sirona) exhibited a pale yellowish tint. During the development of the powder blend, the sintered material appeared brown, which is likely attributable to the oxidation of trace Fe^2+^ impurities present in the raw montmorillonite; however, this colour change was limited to the powder stage and did not translate into discolouration of the set material. Importantly, no visually detectable colour change of the BHA‐MMT composite was observed following short‐term immersion in water, sodium hypochlorite, or bovine blood. Colour assessment was limited to qualitative visual observation and represents a study limitation; quantitative spectrophotometric analysis was beyond the scope of this work. This behaviour may be attributed, at least in part, to the use of zirconium dioxide as the radiopacifier, which has been widely reported to exhibit improved colour stability compared with bismuth oxide (Cutajar et al. 2011; Tour Savadkouhi and Fazlyab 2016). By contrast, ProRoot MTA demonstrated visible discolouration following 24 h immersion in sodium hypochlorite and bovine blood, consistent with previous reports linking this effect to the presence of bismuth oxide and its interaction with irrigants and blood products (Camilleri 2014; Camilleri et al. 2020; Marciano et al. 2015). While the present findings indicate favourable short‐term colour stability, intrinsic tooth discolouration is governed by more complex long‐term interactions within dentine. Long‐term intrinsic tooth discolouration is influenced by the overall material composition rather than by the radiopacifier alone. Accordingly, contributions from other compositional components must also be considered. Montmorillonite clays are reported to contain trace Fe^2+^/Fe^3+^ species, and non‐lattice iron ions may leach under alkaline conditions and potentially contribute to intrinsic discolouration over extended periods, as described previously (Camilleri 2014). Although no discolouration was observed under the short‐term conditions evaluated in this study, these findings do not fully replicate the complex mechanisms of long‐term intrinsic discolouration within dentine. Such effects have been demonstrated using extracted human teeth models, where composition‐dependent discolouration develops over extended periods of evaluation (Palma et al. 2019, 2020; Marciano et al. 2015).

Radiopacity is an essential requirement for endodontic materials. Based on previous literature and the pilot study, non‐staining zirconium dioxide was selected as the radiopacifier and kept at 30% in the formulation (Cutajar et al. 2011; Tour Savadkouhi and Fazlyab 2016). ISO 6876:2012 recommends a radiopacity equivalent to at least 3 mm of aluminium (Al) for endodontic sealers. All three BHA‐MMT composites exceeded the minimum radiopacity requirement of ISO 6876:2012 (≥ 3 mm Al equivalent) and exhibited values fully compliant with clinical standards for endodontic sealers. However, the control ProRoot MTA exhibited a higher radiopacity compared with the experimental composite due to the presence of bismuth oxide, a much heavier element with many tightly bound inner electrons. These inner electrons absorb and scatter X‐rays more efficiently, making bismuth oxide appear brighter on radiographs compared to the zirconium oxide (atomic number 40) (Sarunket et al. 2022). The radiopacity values of zirconium dioxide–containing commercial products, such as Endosequence BC Sealer (3.834 ± 0.346 mm Al) and AH Plus (6.936 ± 0.462 mm Al), fall within the range reported for contemporary endodontic materials and indicate that the radiopacity of the BHA–MMT composite is clinically acceptable.

The compressive strength of the developed composite material ranged from 19.2 to 23.81 MPa, on par with Dycal, a calcium hydroxide‐based lining cement that has been widely used for many years in the management of caries and pulpotomy procedures (Islam et al. 2023; Edwards et al. 2021; Duncan et al. 2023).

The prolonged setting time of conventional MTA materials, including ProRoot MTA, represents a significant limitation, with an initial setting time ranging from 2.5 to 4.0 h and a complete curing period of up to 21 days (Pushpalatha et al. 2022). An optimal setting time for endodontic materials should balance sufficient working time for clinical manipulation and a fast enough setting time to minimise risks, such as material leakage and tissue irritation (Juntha et al. 2024; Allan et al. 2001). The setting time observed for the BHA‐MMT material closely aligns with that of Biodentine (Septodont, Paris, France), a well‐established HCSC with an initial setting time of approximately 12 min, which represents a notable improvement over other HCSCs (Pushpalatha et al. 2022). While statistical comparisons were limited by the small sample size (*n* = 3), the observed differences support the faster‐setting behaviour of the BHA–MMT composite. In addition to its setting time, Biodentine is widely regarded as a contemporary clinical reference material because of its favourable handling characteristics and early mechanical stability reported in the literature (Camilleri et al. 2013). Although direct comparison of handling properties and early mechanical performance was beyond the scope of this proof‐of‐concept study, the observed setting behaviour and compressive strength range of the BHA–MMT composite are consistent with its intended use as a liner‐type, bioactive endodontic material. Although Vicat testing is widely used, the Gilmore needle may better reflect clinical handling of liner‐type materials. Direct comparative evaluation against more contemporary calcium silicate–based materials, such as Biodentine, will be undertaken in future studies (Camilleri 2014). The BHA‐MMT composite is a hydraulic cement with a hydrated crystalline form, which indicates several changes in the structure. FTIR data confirmed the presence of hydroxyapatite in the hydrated form. Peaks at 1450 and 1421 cm^−1^ are typically associated with calcite and carbonate‐substituted HA, and the peaks at 874 and 775 cm^−1^ also suggest the formation of carbonate‐substituted HA, calcite, or both. The calcite formation is also confirmed by XRD reflective angles 29.4°, 36.0°, and 39.5°. Carbonate‐substituted HA, also known as carbonate apatite, is similar in structure to biological bone apatite (Sato et al. 2025; Ishikawa and Hayashi 2021). Calcium silicate hydrate‐related features were suggested by Si–O stretching bands at 1042 and 1090 cm^−1^, which overlap with phosphate (${\mathrm{PO}}_{4}^{3-}$) stretching bands of apatite, leading to possible overlap. A clear Si–O–Si bending band is also visible at 450 cm^−1^. These silicate‐related vibrations are consistent with the formation of calcium silicate hydrate (C–S–H), the principal phase responsible for the setting and hardening of the BHA–MMT material. XRD patterns of the BHA‐MMT showed Ca(OH)_2_ as a byproduct of silicate hydration, similar to MTA. This appeared at 18° and 34°, supported by the FTIR peak between 3640 and 3645 cm^−1^. Notably, the presence of Ca(OH)_2_ raises the pH and releases calcium ions that support bioactivity. Another product is ettringite, a calcium aluminate sulphate mineral, at 22° and 15°. This is likely formed from MMT and sulphates. In addition, the presence of MMT, Calcium silicates, hydroxyapatite and ZrO_2_ is evident from the FT‐IR and XRD patterns (Figure 4).

Although the FT‐IR and XRD analyses confirmed the presence of the main phases and hydration‐related products, clear identification of calcium silicate hydrate (C–S–H) is challenging in the present material. This is largely due to the relatively low calcium silicate content (≤ 22.75 wt%) compared with conventional HCSCs, as well as the dominance of highly crystalline phases such as montmorillonite, hydroxyapatite, and zirconium dioxide. In addition, C–S–H is poorly crystalline and its characteristic features tend to overlap with other silicate‐ and phosphate‐related signals, while the sintering step used in this study further distinguishes the system from conventional non‐sintered HCSCs. Taken together, the XRD and FTIR findings are consistent with calcium silicate hydration, although *direct confirmation of C–S–H would require additional characterisation*.

The pH of endodontic materials also contributes to their performance in several ways. The pH has direct antibacterial activity, neutralises the acidic environment in inflammatory tissues, supports bone repair and mineralisation by enzyme activation, provides local ions, releases growth factors from dentine and other hard tissues, and improves setting time, sealing and stability of the material (Pawińska et al. 2017; Kahler et al. 2017). ProRoot MTA has an initial moderate alkaline pH of 10.2, which increases up to 12.5 after 3 h (Schmitt et al. 2001; Torabinejad et al. 1995). The composite materials demonstrated pH levels of 9.5–10.27 after 3 h. At pH 9.5, materials exhibit relatively low antibacterial activity due to the influence of pH (Siqueira Jr and Lopes 1999). However, after 24 h, all materials reached a pH above 11.5, which was maintained up to 21 days. A strongly alkaline pH indicates potent antibacterial activity, which is potentially beneficial for pulp capping materials to reduce reinfection and promote pulp healing.

In endodontic materials, solubility refers to the maximum permitted mass loss of a set material after immersion in water and is a critical parameter for maintaining seal integrity and long‐term performance. A highly soluble endodontic material can create gaps and voids at the material‐tooth interface, leading to microleakage and secondary infections (Poggio et al. 2010). Notably, all three BHA–MMT composites satisfied the ISO 6876:2012 solubility requirement (≤ 3% mass loss after immersion), ensuring long‐term seal integrity and compliance with established standards.

Heavy metals were once considered a major concern for MTA used in clinical practice. MTA is composed of materials similar to Portland cement, but is more refined and manufactured under stricter guidelines (Schembri et al. 2010). Studies performed to determine heavy metal content in White MTA (Dentsply, Tulsa Dental Products, Tulsa, OK) and MTA Angelus (Angelus Soluções Odontológicas, Londrina, Brazil) reported a heavy metal content, specifically for arsenic, higher than limits allowed in ISO 9917‐1 (Monteiro Bramante et al. 2008). However, newer MTA formulations, such as for ProRoot MTA, also report the presence of heavy metals in trace amounts; nonetheless, these concentrations remain well within permissible limits (Chang et al. 2011). ICP‐MS elemental analysis demonstrated that the concentrations of heavy metals in both the BHA–MMT composite and ProRoot MTA remained well below the permissible limits specified in ISO 9917‐1:2003, particularly for cadmium and arsenic.

The ICP‐MS analysis used *aqua regia* to digest the samples; however, this method did not fully digest elements such as zirconium, silicon, and aluminium compounds in these specimens, and therefore they were not accurately represented in the analytical data. This limitation may have resulted in biased values, and the use of a more sophisticated digestion system could have provided a more accurate and complete elemental analysis.

Saos‐2 cells are commonly used to evaluate the biocompatibility of dental materials such as endodontic materials, because they mimic bone‐forming cells important for periapical healing. They are easy to culture and provide more consistent results than primary pulpal or periodontal cells, which are harder to obtain and more variable (Ratnayake et al. 2023, 2020, 2017; Yong et al. 2022). In addition, Saos‐2 cells are widely employed for the assessment of mineralisation‐related bioactivity of calcium silicate–based endodontic materials, including alkaline phosphatase activity and mineralised nodule formation, providing a reproducible platform for initial biological screening (Zordan‐Bronzel et al. 2019; Tanomaru‐Filho et al. 2017). However, Saos‐2 cells do not fully replicate the biological environment of the dentine–pulp complex, and the present findings should therefore be interpreted as indicative of early bioactivity rather than definitive pulp‐specific remineralisation. A limitation of the cell‐based biological assays in this study is the relatively small sample size typical of in vitro screening experiments, which restricts formal verification of parametric assumptions; therefore, the associated statistical analyses should be interpreted as exploratory.

All three BHA‐MMT composites supported Saos‐2 cell viability. ProRoot MTA samples initially showed good cell viability at 24 h. However, after 72 h, the number of dead cells increased compared to the BHA‐MMT composites and this may be due to the higher alkalinity and the absence of the bioactive hydroxyapatite. The proliferation assay showed increased cell number over 72 h for all materials (Figure 8). After the 24 h period, B20 variation showed the highest cell proliferation rate, followed by B25 and ProRoot MTA. Potentially this could be due to several reasons, including the mild alkalinity due to Ca^2+^ and ${\mathrm{PO}}_{4}^{3-}$ in the B20 and B25 variation. In the B20 variation, the silicate–calcium carbonate–sulphate phase may release more Ca(OH)_2_ from the onset. This facilitates the buffering of the pH and stimulates the cells. In addition, BHA has a surface chemistry similar to bone hydroxyapatite supporting immediate protein adsorption and this may explain the higher early cell growth. However, after 48 and 72 h, M20 showed higher cell proliferation, and this may be linked to the MMT. MMT needs time to exfoliate, hydrate, and expose its silicate layers before becoming bioactive. In the longer term, both BHA and MMT may work in combination. This synergy supports bioactivity and promotes cell proliferation. However, there may be other contributing factors, and further studies are needed to determine their mode of action (Posada‐Lotero et al. 2025; Jamil et al. 2022; Peramune Arachchilage et al. 2024).

Remineralisation is a potentially beneficial function of endodontic sealers that is largely absent in current sealers, as it repairs demineralised dentine through hydroxyapatite formation, which can strengthen the dentine and improve the seal at the dentine–material interface (Baras et al. 2020). The present study demonstrates HA formation and its surface deposition on the BHA–MMT composites. The deposited HA appeared as acicular spherulites, resembling the spherulitic structures reported in several previous studies (Gandolfi et al. 2010). Furthermore, Energy‐Dispersive X‐ray Spectroscopy (EDX) spectral data confirmed the presence of HA, revealing a Ca/P ratio of approximately 1.67, characteristic of stoichiometric HA.

SEM analysis showed that the M20 material displayed extensive apatite coverage across the surface, whereas the B20 specimen showed the least mineral deposition. Comparatively, the B25 and MTA specimens exhibited relatively moderate levels of apatite on the composite surface. Although the difference in MMT content is only 5% between M20 and the other two composite specimens, the higher MMT proportion appears to influence apatite formation positively. These findings indicate synergistic bioactivity from BHA (immediate protein adsorption and apatite similarity to bone) and MMT through its negative surface charge, aiding mineral nucleation (Šupová 2024); however, this mechanism remains plausible and requires confirmation via ion release profiles (Ca, P, Si) and zeta potential measurements. HA formation was reported to have the greatest surface coverage on ProRoot MTA, followed by MTA Fillapex and GuttaFlow Bioseal (AL‐Askary et al. 2025). In contrast, the present findings demonstrate that the BHA–MMT composites exhibited substantially higher HA coverage than ProRoot MTA, indicating that the BHA–MMT composites may exhibit improved remineralisation potential compared with ProRoot MTA, MTA Fillapex, and GuttaFlow Bioseal (AL‐Askary et al. 2025).

The SEM observations of HA deposition and the Alizarin Red staining assay results are consistent, indicating calcium deposition, with the M20 specimen showing the highest deposition level from the cultured cells. Alizarin Red staining indicates the degree of mineralisation in osteoblast‐like Saos‐2 cells that have been induced to differentiate and deposit bone matrix, which subsequently becomes mineralised. Alizarin Red staining confirmed calcium deposition across all tested specimens, with the M20 specimen exhibiting the highest calcium deposition. Although the precise factors contributing to this enhanced apatite formation and calcium deposition require further investigation, it is plausible that the higher MMT content in the M20 formulation played a significant role. Accordingly, confirmation of these findings in clinically relevant stem cell models remains an important focus of future work. To robustly support such claims, future studies should employ clinically relevant pulp models such as dental pulp cells (DPCs) or dental stem cell models, such as human dental pulp stem cells (hDPSCs) or stem cells from the apical papilla (SCAP), combined with odontogenic marker‐based analyses (e.g., ALP, OCN, DSPP), as demonstrated in previous pulp‐focused biological assessments (Sequeira et al. 2018, 2021).

Moreover, given the novel composition of the present material, incorporating montmorillonite, bovine‐derived hydroxyapatite, and a sintered multi‐phase matrix, its ion release behaviour cannot be assumed to follow conventional calcium silicate hydrate–dominated systems and is likely influenced by additional physicochemical interactions arising from these components.

Calcium silicate–based endodontic materials have evolved markedly in recent years, with newer formulations offering improved handling, faster setting, and greater clinical convenience. As a result, these materials are now widely used in contemporary endodontic practice. Nevertheless, fundamental performance requirements such as bioactivity, physicochemical stability, and biocompatibility remain central to material evaluation and continue to guide the development of new systems.

Within this context, the present study should be regarded as an early‐stage, proof‐of‐concept investigation of a novel composite material designed to meet these core requirements. The developed BHA–MMT formulation represents a foundational material platform rather than a finished clinical product. Further optimisation is required, and direct comparison with currently used materials will be essential in future studies to more clearly establish its clinical relevance.

The interaction between calcium silicate–based materials and the adhesive interface is clinically important, as their alkaline environment, ongoing moisture release, and water sorption can adversely affect adhesive polymerisation and, consequently, the durability of resin‐based restorations. Previous studies have demonstrated that restorative timing and dentine pretreatment significantly influence bond strength to calcium silicate cements (Palma et al. 2021; Marques et al. 2025), while recent evidence has further highlighted the importance of irrigation protocols and adhesive strategies in achieving a stable adhesive interface (Marques et al. 2024; Xavier et al. 2021). Although bond strength testing was beyond the scope of this initial proof‐of‐concept study, systematic evaluation of the adhesive interface will be essential in future investigations once the material formulation has been finalised.

Within the scope of the present study, the BHA–MMT composite is best positioned for vital pulp treatment applications, including direct and indirect pulp capping and use as a liner or base beneath a definitive restoration, as well as perforation repair and root‐end filling procedures, where sealing ability, bioactivity, alkaline pH, radiopacity, and physicochemical stability are more critical than high load‐bearing capacity. For these applications, moderate compressive strength, along with dimensional stability, is clinically appropriate. These potential applications warrant further targeted evaluation in future studies.

Beyond laboratory performance, material accessibility, cost, and clinical practicality remain critical determinants of adoption in contemporary endodontic practice.

Recent multinational surveys of contemporary endodontic practice reveal considerable variation in the availability, cost, and clinical uptake of advanced regenerative and bioactive materials across different regions. Findings from large‐scale surveys by BA‐Hattab et al. (2025) indicate that material cost, ease of handling, and accessibility continue to play a major role in clinical decision‐making, particularly in low‐ and middle‐income settings. The development of this bioactive calcium‐based material has the potential to reduce practical barriers to broader clinical use while preserving favourable biological properties.

The developed BHA–MMT material was formulated using readily available raw materials, including bovine‐derived hydroxyapatite obtained from waste bovine bone and a naturally occurring montmorillonite clay. Compared with fully synthetic alternatives, these components are relatively readily available as raw materials and may help reduce material input costs during formulation. In the New Zealand context, the use of bovine bone as an industrial by‐product, together with locally sourced clay minerals, is relevant to material selection and may support scalability and sustainability during material development. The broader translation of animal‐derived biomaterials also requires consideration of regulatory, ethical, and clinical contexts. The use of bovine‐derived hydroxyapatite is not limited to New Zealand but is regulated under medical device frameworks in Europe, North America, and Asia, and it has an established history of clinical use in approved dental graft materials such as Bio‐Oss and cerabone. Nevertheless, patient preference remains important, and bovine hydroxyapatite can be directly substituted with synthetic hydroxyapatite for patients who wish to avoid animal‐derived materials.

Furthermore, calcium silicate–based materials such as Biodentine and other bioceramic systems represent a later stage in the development of MTA‐derived products, reflecting extensive optimisation and clinical validation. While the compressive strength of the present material is lower than that of these established formulations, this property is not critical for its intended clinical use. The material was developed as a proof‐of‐concept system for applications such as direct and indirect pulp capping, perforation repair, and root‐end filling, where rapid setting, bioactivity, and remineralisation potential are of greater clinical relevance than high load‐bearing capacity.

## 5 Conclusions

The use of HA in endodontic composites has been limited by weak mechanical properties. This study shows that combining HA with montmorillonite can address these challenges. The composites developed here met ISO standards for radiopacity, colour stability, and solubility, and maintained an alkaline pH that may contribute to antimicrobial activity.

HA improved the biocompatibility compared with ProRoot MTA, while higher montmorillonite content enhanced apatite formation and calcium deposition. These features are closely linked to dentine healing and lesion sealing. The compressive strength was also similar to that of calcium hydroxide, which is already widely used as a pulp‐capping agent, suggesting immediate clinical relevance.

The use of waste bovine bones as a source of HA represents an efficient materials‐utilisation strategy and highlights the potential to incorporate readily available resources into the development of bioactive endodontic composites. Such cost efficiency enables the development of affordable, high‐quality composite materials from naturally sourced products and materials for endodontic applications. Overall, the results highlight the promise of BHA–MMT composites as bioactive pulp‐capping materials, with further optimisation and in vivo studies needed to support clinical applicability.

## Author Contributions

Conceptualisation: P.A.A.S.P.K., P.R.C., P.C., G.D., H.F.D. and J.R. Data curation: P.A.A.S.P.K. Formal analysis: P.A.A.S.P.K. Investigation: P.A.A.S.P.K. Methodology: P.A.A.S.P.K. Project administration: J.R. Resources: J.R., P.R.C. and G.D. Supervision: J.R., P.R.C., P.C., H.F.D., M.G. and G.D. Validation: P.A.A.S.P.K. Visualisation: P.A.A.S.P.K. Writing – original draft preparation: P.A.A.S.P.K. and J.R. Writing – review and editing: P.R.C., P.C., H.F.D., M.G., G.D. and J.R. Funding acquisition: J.R. and P.R.C.

## Funding

This work was supported by University of Otago, 10.13039/100008247.

## Conflicts of Interest

The authors declare no conflicts of interest.

## Supporting information


**Figure S1:** *From: Nagendrababu V, Murray PE, Ordinola‐Zapata R, Peters OA, Rôças IN, Siqueira JF Jr, Priya E, Jayaraman J, Pulikkotil SJ, Camilleri J, Boutsioukis C, Rossi‐Fedele G, Dummer PMH (2021) PRILE 2021 guidelines for reporting laboratory studies in Endodontology: a consensus‐based development. *International Endodontic Journal* May 3. doi:10.1111/iej.13542.

**Figure S2:** Representative samples showing the colour of the fully set BHA‐MMT composites compared with ProRoot MTA.

## Data Availability

The data that support the findings of this study are available from the corresponding author upon reasonable request.
