# Supplementary material for: Development of a Nano‐Hybrid Composite Using Bovine Hydroxyapatite and Montmorillonite for Endodontic Applications—An In Vitro Study
Source: Int Endod J. 2026 Apr 15;59(9):1936–53. doi: 10.1111/iej.70163 (PMC13462442; doi:10.1111/iej.70163)
Supplement: Supplementary file 1 [file IEJ-59-1936-s001.docx]

**PRILE 2021 Flowchart**

RATIONALE/JUSTIFICATION The true impact of incorporating bovine-derived hydroxyapatite (BHA) and montmorillonite (MMT) nanoclay on the mechanical and biological performance of endodontic composites remains largely unexplored. Understanding whether this hybrid approach can enhance strength and biocompatibility while overcoming the limitations of calcium silicate–based materials such as ProRoot MTA is crucial for assessing its clinical potential.

AIM/HYPOTHESIS This study aimed to develop and characterise a bovine-derived hydroxyapatite (BHA) and montmorillonite (MMT) nano-clay-based hybrid composite material (BHA-MMT), incorporating zirconium oxide as a radiopacifier and calcium silicate phases as setting agents.

ETHICAL APPROVAL Not Applicable

SAMPLES Bovine hydroxyapatite, Montmorillonite, Zirconium oxide, Tricalcium silicate, Dicalcium silicate, Calcium carbonate, Calcium sulphate and instruments such as Ball-Mill, Oven, and FTIR, Scanning electron microscope with Energy-dispersive X-ray spectroscopy

EXPERIMENTAL AND CONTROL GROUPS, INCLUDE INDEPENDENT VARIABLES Bovine hydroxyapatite and montmorillonite nanoclay were combined with zirconium oxide, setting agents, and modifiers to produce three composites—B20, B25, and M20. The resulting cements were characterized using XRD, FTIR, SEM/EDX, and ICP-MS, and evaluated through mechanical, physical, and biological assays, including cell viability, proliferation, and remineralization.

OUTCOME(S) ASSESSED, INCLUDE DEPENDENT VARIABLES The study evaluated the physicochemical, mechanical, biological, and remineralization properties of the developed BHA–MMT composites, with dependent variables including setting time, pH, solubility, strength, radiopacity, cell viability, and mineralisation. Tests were conducted in triplicate or with higher sample numbers as a quantitative in vitro study.

METHOD USED TO ASSESS THE OUTCOME (S) AND WHO ASSESSED THE OUTCOME(S) Outcomes were assessed using standard methods including Vicat apparatus, pH meter, radiography, XRD, FTIR, ICP-MS, and cell-based assays (MTS and Alizarin Red). All tests were conducted by the research team under academic supervision.

RESULTS The BHA–MMT composite variants (B20, B25, and M20) exhibited acceptable physicochemical properties, alkaline pH, good biocompatibility, and enhanced mineralisation potential, with adequate compressive strength and scope for further improvement through optimization.

CONCLUSION(S ) The BHA–MMT composites exhibited favourable physicochemical and biological properties, meeting ISO standards and showing potential as a cost-effective alternative to existing materials. Further in-vivo validation is needed to confirm clinical suitability

FUNDING DETAILS This research was funded by the University of Otago Research Grant (UORG), Grant No. 12056801RJD

CONFLICT OF INTEREST The authors declare no conflict of interest related to this study

**SUPPLEMENTARY FIGURE S1.** ***From: Nagendrababu V, Murray PE, Ordinola-Zapata R, Peters OA, Rôças IN, Siqueira JF Jr, Priya E, Jayaraman J, Pulikkotil SJ, Camilleri J, Boutsioukis C, Rossi-Fedele G, Dummer PMH (2021) PRILE 2021 guidelines for reporting laboratory studies in Endodontology: a consensus-based development. *International Endodontic Journal* May 3. doi: 10.1111/iej.13542.** [**https://onlinelibrary.wiley.com/doi/abs/10.1111/iej.13542**](https://onlinelibrary.wiley.com/doi/abs/10.1111/iej.13542)**.**

**For further details visit:** [**http://pride-endodonticguidelines.org/prile**](http://pride-endodonticguidelines.org/prile)
