# Supplementary material for: Development of a Nano‐Hybrid Composite Using Bovine Hydroxyapatite and Montmorillonite for Endodontic Applications—An In Vitro Study
Source: Int Endod J. 2026 Apr 15;59(9):1936–53. doi: 10.1111/iej.70163 (PMC13462442; doi:10.1111/iej.70163)
Supplement: Supplementary file 2 [file IEJ-59-1936-s002.docx]

**SUPPLEMENTARY FIGURE S2.** **Representative samples showing the colour of the fully set BHA-MMT composites compared with ProRoot MTA.**
